# Mind the Gap! A Multilevel Analysis of Factors Related to Variation in Published Cost-Effectiveness Estimates within and between Countries

**DOI:** 10.1177/0272989X15579173

**Published:** 2016-01

**Authors:** Christian E. H. Boehler, Joanne Lord

**Affiliations:** Institute for Prospective Technological Studies, Joint Research Centre–European Commission, Seville, Spain (CEHB); Health Economics Research Group, Brunel University, Uxbridge, UK (JL)

**Keywords:** economic evaluation, transferability, exchangeability, multilevel statistical modeling, statins, cardiovascular disease

## Abstract

***Background.*** Published cost-effectiveness estimates can vary considerably, both within and between countries. Despite extensive discussion, little is known empirically about factors relating to these variations. ***Objectives.*** To use multilevel statistical modeling to integrate cost-effectiveness estimates from published economic evaluations to investigate potential causes of variation. ***Methods.*** Cost-effectiveness studies of statins for cardiovascular disease prevention were identified by systematic review. Estimates of incremental costs and effects were extracted from reported base case, sensitivity, and subgroup analyses, with estimates grouped in studies and in countries. Three bivariate models were developed: a cross-classified model to accommodate data from multinational studies, a hierarchical model with multinational data allocated to a single category at country level, and a hierarchical model excluding multinational data. Covariates at different levels were drawn from a long list of factors suggested in the literature. ***Results.*** We found 67 studies reporting 2094 cost-effectiveness estimates relating to 23 countries (6 studies reporting for more than 1 country). Data and study-level covariates included patient characteristics, intervention and comparator cost, and some study methods (e.g., discount rates and time horizon). After adjusting for these factors, the proportion of variation attributable to countries was negligible in the cross-classified model but moderate in the hierarchical models (14%−19% of total variance). Country-level variables that improved the fit of the hierarchical models included measures of income and health care finance, health care resources, and population risks. ***Conclusions.*** Our analysis suggested that variability in published cost-effectiveness estimates is related more to differences in study methods than to differences in national context. Multinational studies were associated with much lower country-level variation than single-country studies. These findings are for a single clinical question and may be atypical.

Decisions about the provision and reimbursement of health technologies are increasingly informed by consideration of cost-effectiveness. Ideally, deliberations would always be informed by a bespoke economic evaluation, tailored to the specific decision problem of interest, using a preferred methodological framework and integrating best evidence of effectiveness with local data and assumptions on the epidemiological, health care, and socioeconomic context. In practice, decision makers do not always have the resources to commission such exemplary economic evidence, and even bodies that invest heavily in analysis do not have access to a bespoke decision model to inform every decision. For example, although the Technology Appraisal Committees at the National Institute for Health and Care Excellence in England do usually have access to a bespoke model, the Clinical Guidelines and Public Health committees often do not. In such cases, rather than not considering economic evidence at all, decision makers may seek to transfer or adapt cost-effectiveness estimates from other contexts. However, results of published economic evaluations often vary, and it is hard to know which estimates to rely on and whether and how they might be combined.

Quantitative pooling of cost-effectiveness results is not generally recommended because of concerns over heterogeneity of methods, decision perspective, and intervention context.^1^ The Cochrane Handbook emphasizes that the preferred method to summarize economic evidence is through tabulation of the characteristics and results of included studies, supplemented by a narrative summary.^2^ The GRADE collaboration has taken a similar position.^3^ Anderson and Shemilt^4^ go further and argue that attempts to pool results from multiple economic studies are fundamentally misguided and that the (only) good reasons for conducting reviews of economic studies are 1) to inform the development of a bespoke model, 2) to identify the most relevant study to transfer or adapt when de novo modeling is not possible, and 3) to understand the key economic tradeoffs and causal relationships relevant to a decision. Shemilt and others^5^(p8) suggest that multivariate meta-regression may allow *“*the effects of multiple explanatory factors to be investigated simultaneously,’’ but that “techniques to explore the impact of factors likely to explain variation in estimates of resource use, costs and effects between studies remains under-explored for economic data.”

There is now a considerable literature on the transferability or generalizability of economic evaluations of health care technologies, and many potential factors have been suggested that may account for variability between estimates.^6,7^ However, these factors are often fuzzy or difficult to measure, and little is known empirically about their relative importance. Understanding the factors relating to variability in published estimates of cost-effectiveness is not just of academic interest to health economists but may potentially help decision makers to decide what aspects of published economic evaluations are most important when deciding whether or not to transfer or adapt results to their context and, if so, which of the available estimates is most relevant for them.

This article aims to explore variation in estimates of incremental costs (ΔC) and incremental effects (ΔE) between studies and countries using a multilevel-modeling (MLM) approach. We were interested in the extent of intercountry variation in cost-effectiveness estimates, after controlling for population, intervention, and methodological differences within and between studies. We also sought to explore whether between-country variation could be explained by any measureable national characteristics (such as gross domestic product [GDP] per head or expenditure on health care).

MLM is not new to health economics.^8,9^ The method has been applied to analyze individual patient data (IPD) from economic evaluations conducted alongside multinational randomized controlled trials (RCTs),^6,10–16^ cluster randomized trials,^17–19^ and multicentre observational studies.^20–23^ Studies have used resource use,^20^ cost,^11,16,20,22^ or net monetary benefit^6,12,17,21^ as a response variable or estimated incremental costs (ΔC) and incremental effects (ΔE) simultaneously in a bivariate framework.^10,13–15,17,19,21^ A 2-level hierarchical data structure is common, with patient data grouped in centers,^6,13,16,20–23^ patient clusters,^17–19^ or countries.^10,11,14,15^ Our contribution extended this approach, drawing from research on school performance,^24^ to use 3-level hierarchical and cross-classified model structures with published cost-effectiveness estimates grouped within studies and countries. The hierarchical model structure requires that studies are nested within countries—that each study provides estimates for 1 and only 1 country. The cross-classified model allows the inclusion of results from multinational studies, which report estimates for more than 1 country. We compare the results from these model structures.

We applied our models to a set of published results derived from a systematic review of economic evaluations on the use of statins for the prevention of cardiovascular disease (CVD). Statins were deliberately chosen as we knew that they had been extensively studied and that many cost-effectiveness estimates would be available for many countries, thus increasing power for our analyses. The results are therefore illustrative and should not be seen as typical for other interventions or disease areas. The studies that we used included both trial-based economic evaluations (based on an analysis of IPD from a single RCT) and model-based evaluations (in which estimates of treatment effects from a trial or meta-analysis of trials were combined with parameter estimates from other sources in a decision model). From each study, we extracted pairs of (ΔC, ΔE) estimates for the base case analysis and for each reported subgroup and deterministic sensitivity analysis. Thus, each study could yield more than 1 data point for analysis, providing information about how the factors defining the different subgroup or sensitivity analyses contributed to variation in the study estimates. This use of published cost-effectiveness estimates allowed us to investigate the impact of methodological differences between studies, which has been previously identified as an important source of variability,^25^ alongside intercountry variation.

Like all MLM, our approach relies on an assumption of exchangeability,^26^ that is, that there are “no a priori reasons why one jurisdiction may have more or less favourable measures of costs or cost-effectiveness than another.”^27(p413)^ Within the MLM framework, we can model exchangeability while also controlling for variation within studies, between studies, and between countries through the assumption of conditional independence.^10,28,29^ This allows quantification of the impact of variability factors on data, study, and country level. However, a fundamental difference between our study and the above applications of MLM was the unit of analysis: previous studies analyzed data for individual patients, while we analyzed summary estimates of (mean) incremental costs (ΔC) and health effects (ΔE) reported in published economic evaluations. Our analyses may be seen as a form of meta-regression but with the difference that it was not possible to incorporate the precision of each published estimate of ΔC and ΔE. Most studies in our data set (61 out of 67) relied on decision modeling rather than observations from IPD, and estimates of precision based on sampling variation are not available for these studies. This issue is addressed in the Discussion section below.

In the following Methods section, we describe how the data set was obtained from a systematic review, specify alternative model structures, and explain how potential explanatory variables were derived from the literature. In the Results, we describe the statins data set, compare the fit of the alternative model specifications without covariates, investigate how the level of country variation changes with the inclusion of covariates at data and study level, and finally assess some covariates at country level. The Discussion highlights the strength and weaknesses of our approach, puts our findings in the context of existing literature, and outlines potential policy implications and areas for further research.

## Methods

### Derivation of the Data Set

We conducted a systematic literature review to identify cost-effectiveness estimates for statins in the prevention of CVD. A sensitive search strategy was used to identify as many economic evaluations for as many countries as possible. A search of electronic databases (OVID, PubMed, SCOPUS, Web of Science, HEED, CRD, NHS EED, Biosis, EBSCO, and Cochrane) was conducted using search terms from a published review.^30^ Reviews were also hand searched to identify any additional studies. Studies were included if they compared a statin with “doing nothing,”“another statin,” or “the same statin in a different dosage” in an adult population and if point estimates of mean incremental costs ΔC and effects ΔE could be retrieved, with effectiveness measured either in life-years saved or quality-adjusted life-years (QALYs). Studies using trial-based IPD and decision analytic modeling studies were included. Studies that adapted published results to other countries were included if results were not simply currency-adjusted equivalents of the original data. We did not apply further study quality criteria, as we intended to control for study quality using the Quality of Health Economic Studies (QHES) instrument.^31^

Cost-effectiveness estimates were extracted for the base case and for reported subgroup and sensitivity analyses; thus, each study contributed 1 or more point estimates of (ΔC, ΔE). Each data point was associated with a target country, but some multinational studies reported estimates for more than 1 country. Local currencies were transferred to Pounds Sterling using purchasing power parities and updated to 2010 using country-specific GDP deflators.^32,33^ For Eurozone countries, historic currencies were converted to Euros using irrevocable conversion rates.^34^

### Definition of Covariates

Potential explanatory variables were identified from a list of 77 variability factors suggested in previous publications.^6,7^ We coded about 200 measurable indicators of these factors in a data abstraction form and applied this on a pilot data set of 16 studies. Variables that were not frequently reported or did not vary between studies were dropped, resulting in a final set of 59 variables for which data were extracted from the study reports. Finally, country-level variables were added from databases of international statistics.^35,36^

The final list of 71 candidate variables is shown in Supplementary Appendix A. At the data level, there were measures of patient and disease characteristics, intervention and comparator characteristics, and methods that varied within studies (e.g., discount rates that were often tested in sensitivity analysis). At the study level, there were some general characteristics (e.g., language and funding source), methods that varied only between studies (e.g., whether the study was trial or model based), and indicators of study quality (the QHES score^31^). At the country level were indicators of national health care finance and resource, demographic, and epidemiological characteristics.

### Model Specifications

Multilevel statistical modeling was used to investigate factors likely to explain variation in published cost-effectiveness information.

#### Hierarchical model

**Figure 1:**
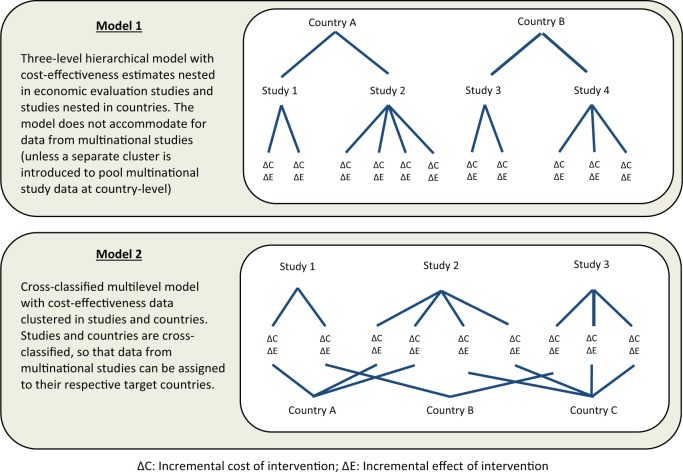
Developing multilevel models to reflect complex structures in a data set of published cost-effectiveness estimates (model descriptions and unit diagrams).

First consider the situation in which each study provides estimates for 1 and only 1 country. In this case, there is a strictly hierarchical data structure, with published estimates of ΔC and ΔE (level 1) clustered in studies (level 2) and with studies clustered in countries (level 3). This is illustrated in the top section of Figure 1.

A bivariate model specification was used to recognize the correlation between the response variables ΔC and ΔE, while allowing them to have different covariates and coefficients.^10,13^ This also has the advantage that the regression can be run without assuming a particular value for the cost-effectiveness threshold λ. Let *y_lijk_* be the incremental cost and *y*_2*ijk*_ be the incremental effect for the *i*-th point estimate reported in the *j*-th study for the *k*-th country. The model consisted of a correlated pair of linear regression equations, with explanatory variables and error terms on each level of the data hierarchy:

$$y_{dijk}=\beta_{0d}+\beta_{1d}x_{1dijk}+\beta_{2d}x_{2djk}+\beta_{3d}x_{3dk}+v_{dk}+u_{djk}+e_{dijk}d=1,2$$

For each response variable (*y_dijk_, d* = 1,2), there are 3 vectors of covariates: *x_ldijk_* at the data level, *x*_2*djk*_ at the study level, and *x*_3*dk*_ at the country level. The data-level, study-level, and country-level error terms (*e_dijk_, u_djk_*, and *v_dk_*, respectively, *d* = 1,2) are assumed to follow a bivariate normal distribution:

$$\begin{array}{c} \left[\begin{array}{c} v_{1k}\\ v_{2k} \end{array}\right]∼BVN\left(0,\Omega_{v}\right)\;where\Omega_{v}=\left[\begin{array}{cc} \sigma_{v1}^{2}\\ \sigma_{v12} & \sigma_{v2}^{2} \end{array}\right]\\ \left[\begin{array}{c} u_{1jk}\\ u_{2jk} \end{array}\right]∼BVN\left(0,\Omega_{u}\right)\;where\Omega_{u}=\left[\begin{array}{cc} \sigma_{u1}^{2}\\ \sigma_{u12} & \sigma_{u2}^{2} \end{array}\right]\\ \left[\begin{array}{c} e_{1ijk}\\ e_{2ijk} \end{array}\right]∼BVN\left(0,\Omega_{e}\right)\;where\Omega_{e}=\left[\begin{array}{cc} \sigma_{e1}^{2}\\ \sigma_{e12} & \sigma_{e2}^{2} \end{array}\right] \end{array}$$

Note that the MLM framework allows both intercepts and regression slopes to vary randomly, and random slopes could be fitted to test whether the relationships between cost-effectiveness estimates and explanatory variables (for instance, age or baseline cholesterol levels) differ between studies or between countries.^29,37,38^ However, for simplicity, we assumed in this exercise that only intercepts vary randomly, whereas slopes remain fixed across studies and countries. Thus, for example, the difference in the incremental net benefit of statins between older patients and younger patients is assumed to be the same in different countries

#### Cross-classified model

Now consider that some studies might estimate the cost-effectiveness of an intervention for more than 1 target country, for instance, when economic evaluations are conducted alongside multinational trials or when decision models are adapted to several contexts. In this case, the hierarchical data structure breaks down. The resulting cross-classified structure is illustrated in the lower panel of Figure 1.

In MLM notation, parentheses are used to group the subscripts for the cross-classified levels *j* and *k*.^24,29^ Thus, the above model becomes

$$\begin{array}{c} y_{di(jk)}=\beta_{0d}+\beta_{1d}x_{1di(jk)}+\beta_{2d}x_{2dj}+\beta_{3d}x_{3dk}+v_{dk}+u_{dj}+e_{di(jk)}d=1,2\\ \left[\begin{array}{c} v_{1k}\\ v_{2k} \end{array}\right]∼BVN\left(0,\Omega_{v}\right)where\Omega_{v}=\left[\begin{array}{cc} \sigma_{v1}^{2}\\ \sigma_{v12} & \sigma_{v2}^{2} \end{array}\right]\\ \left[\begin{array}{c} u_{1j}\\ u_{2j} \end{array}\right]∼BVN\left(0,\Omega_{u}\right)where\Omega_{u}=\left[\begin{array}{cc} \sigma_{u1}^{2}\\ \sigma_{u12} & \sigma_{u2}^{2} \end{array}\right]\\ \left[\begin{array}{c} e_{1i(jk)}\\ e_{2i(jk)} \end{array}\right]∼BVN\left(0,\Omega_{e}\right)where\Omega_{e}=\left[\begin{array}{cc} \sigma_{e1}^{2}\\ \sigma_{e12} & \sigma_{e2}^{2} \end{array}\right] \end{array}$$

Although this model is implemented with 4 levels in MLwIN software, it is conceptually a 2-level model^29^: the lowest level is required to correlate the pair of linear regression equations for ΔC and ΔE, while cost-effectiveness estimates at level 1 are clustered in studies and countries, where both studies and countries are cross-classified at level 2. A comparable bivariate cross-classified model has been previously used by Goldstein and Sammons^24^ for school performance research.

### Statistics of Interest

The primary measure of model performance was the deviance information criterion (DIC).^39^ This is a Bayesian analogue of the Akaike information criterion suitable for MLMs and combines a measure of model fit (deviance) with an adjustment for model complexity (the number of free parameters). A lower DIC generally indicates a better explanation of the data. Spiegelhalter and others^39^ suggested a rule of thumb for a minimally important DIC difference of 1 to 2 points.

The aim of this study was to explore the extent of intercountry variation in cost-effectiveness estimates in the statin data set, after controlling for differences attributable to study methods, data, and assumptions. Key statistics of interest were therefore the absolute level of variance at the country level ($\sigma_{\mathrm{vd}}^{2}$, *d* = 1,2), and the proportion of total variance attributed to differences between countries: the variance partition coefficient (VPC)^29^:

VPC vd=σvd2/(σvd2+σud2+σed2),d=1,2.

Similar absolute and relative measures of variance were obtained for the data and study levels, but the division of residual (subcountry) variance between these 2 levels is somewhat arbitrary, as it relates to authors’ choices of which parameters to vary in subgroup and sensitivity analyses.

### Estimation Procedure

The models were implemented in MLwiN software (version 2.30, Centre for Multilevel Modelling, Bristol, UK), using Markov chain Monte Carlo (MCMC) estimation with initial iterative generalized least squares to estimate priors when possible.^38,40^ The appropriate length of Markov chains was based on inspection of MCMC trajectories.^40^

We first tested alternative model structures as variance components models without covariates, as recommended in the MLM literature.^29,37,38^ Results are reported for 3 specifications below. First, the cross-classified model was used to accommodate estimates from multinational studies alongside single-country studies. In theoretical terms, this model provided the most appropriate reflection of the data structure. However, we anticipated that between-country variance estimated from single-country and multinational studies might differ due to the use of standardized study protocols and data sources within multinational studies.^25,41^ To explore such effects, we present 2 versions of the hierarchical model: a simple hierarchical model excluding the multinational data and a hybrid hierarchical model incorporating the full data set but with multinational data clustered in a separate group on the country level. The latter hybrid model retains information from multinational studies on data and study level but without confounding country parameters. Our prior expectation was that the proportions of variance at the country level (VPC_*vd*_, *d* = 1, 2) would be higher in the hybrid and fully hierarchical model than in the cross-classified model. Other model specifications were tested, including 2-level models (with only study or country level), but these were clearly inferior to the 3 included models (as indicated by DIC statistics).

After assessing performance of variance components models, we considered the addition of covariates. We first performed extensive collinearity checks and correspondence analyses for categorical data. All continuous variables were centered on their overall means, so that the intercepts represent the predicted ΔC or ΔE for average values for each explanatory variable.^13,29^ We followed a sequential approach by fitting the models from the bottom up as recommended for MLMs.^29,37,38^ We introduced potential covariates in subsets based on a priori reasoning: starting with patient characteristics, regarded as the most fundamental source of variability,^6^ then adding differences in intervention and comparator characteristics, and finally adding covariates that encoded methodological variation within and then between studies. Within each subset, the decision over which covariates to include was based on plausibility of the signs of the coefficients, the magnitude of reduction in DIC, and statistical significance. After specifying multivariate models with covariates on data and study level, we used these models to further explore whether between-country variation could be explained by any measureable national characteristics. The validity of the final models, with covariates at all levels, was investigated graphically by plotting the distributions of residuals at data, study, and country level (caterpillar plots and normal probability plots^29^).

## Results

### Description of Data Set

**Figure 2:**
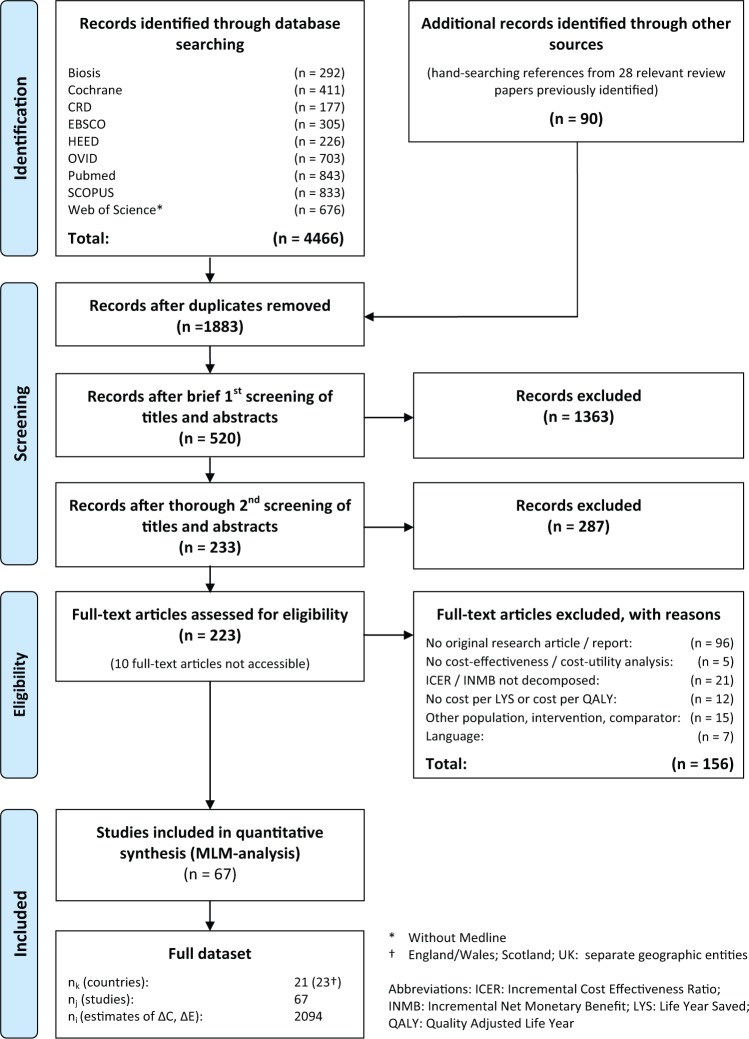
Search algorithm (PRISMA flowchart).

The yield of information from the systematic review is illustrated in the PRISMA flowchart in Figure 2.

**Figure 3:**
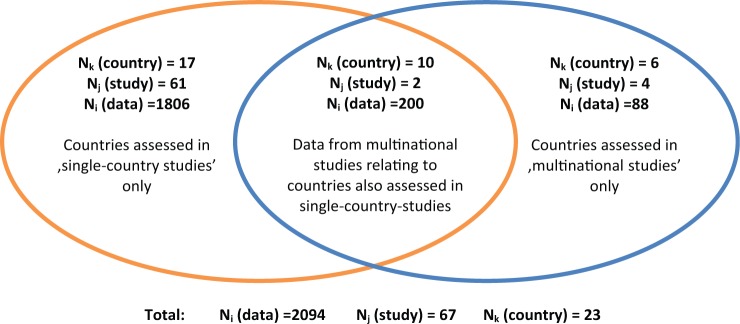
Venn diagram illustrating the nature of the economic evaluation data.

A total of 2094 data points with ΔC, ΔE were extracted from 67 studies across 23 countries (Figure 3). Sixty-one studies provided cost-effectiveness estimates for 1 particular country. The remaining 6 studies were multinational. These 6 studies provided 288 estimates for 16 countries, of which 6 countries were assessed in only multinational studies. Key study characteristics and references are listed in Supplementary Appendices B and C.

Year of publication ranged from 1988 to 2009. Industry was involved in funding 39 studies, whereas funding was unclear for 17 studies. Simvastatin was the most commonly assessed intervention, and “do nothing” was the most common comparator, although 17 studies compared statins. Most studies (61) used a model, while 6 studies made use of IPD. Subgroup analysis was usually performed with respect to age, gender, and pretreatment cholesterol level. QALYs were estimated in 32 studies.

### Variance Components Models

The results for the variance components models are reported in Table 1.

**Table 1:** Variance Components Models (Without Covariates)

Model	Hierarchical Model		Hierarchical Hybrid Model		Cross-Classified Model	
How multinational data were treated on country level?	Data from multinational studies were not included		All data from multinational studies were included and fully assigned to 1 extra group on country level		All multinational study data were included and assigned to their respective target countries	
*n* (Country)	17		17 (+1)		23	
*n* (Study)	61		67		67	
*n* (Data)	1806		2094		2094	
Response Variable	ΔC (SE)	ΔE (SE)	ΔC (SE)	ΔE (SE)	ΔC (SE)	ΔE (SE)
Intercept	6250 (1858)	0.403 (0.123)	6003 (1752)	0.421 (0.124)	6250 (1273)	0.449 (0.085)
$\sqrt{\sigma_{{u0}_{j}}^{2}}$ (Country)	5107	0.381	4680	0.352	1425	0.063
$\sqrt{\sigma_{{u0}_{k}}^{2}}$ (Study)	8564	0.564	8363	0.602	9039	0.648
$\sqrt{\sigma_{c0}^{2}}$ (Data)	10936	0.501	10425	0.559	10418	0.560
Country VPC (%)	11.91	20.31	10.92	15.50	1.06	0.54
Study VPC (%)	33.48	44.54	34.88	45.38	42.49	56.91
Data VPC (%)	54.61	35.15	54.20	39.13	56.45	42.55
DIC	24,573		28,735		28,734	

Note: ΔC = incremental cost; ΔE =incremental effect; DIC = deviance information criterion; SE = standard error; VPC = variance partitioning coefficient.

All models suggested that variability in published cost-effectiveness estimates related more to differences within and between studies than to differences in national context: country-level VPC was no more than 20% in any model. However, the proportion of variance at the country level was much greater when data from multinational studies were excluded or grouped in a separate cluster than when they were attributed to individual countries in the cross-classified model.

**Figure 4:**
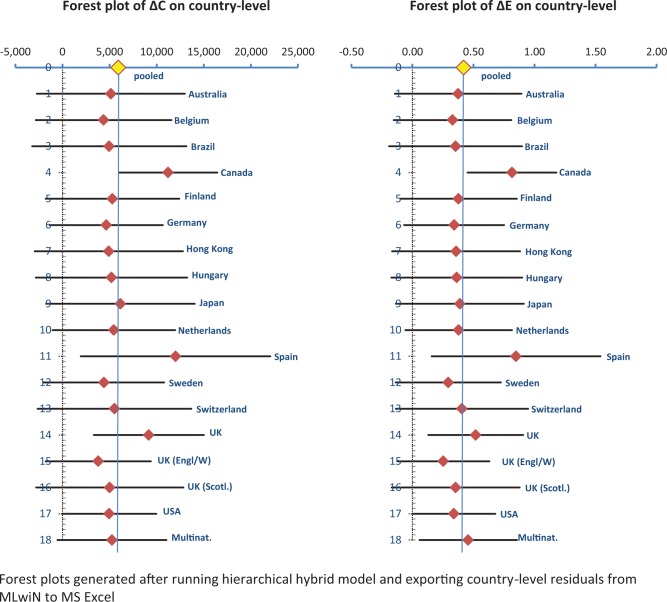
Forest plots of incremental costs and incremental effects on country level.

Forest plots generated from the country-level residuals of the hierarchical hybrid model are shown in Figure 4. This illustrates the means and confidence intervals for ΔC and ΔE for 17 countries as estimated from the 61 single-country studies. The 18th category shows the results for the group of 16 countries estimated from the—comparatively homogeneous—multinational study data, located close to the overall regression mean.

DIC values were very similar for both models that were run on the full data set, although the cross-classified model performed marginally better. The DIC of the hierarchical model is not comparable to the other models as this was run on a subset of the data.

### Multivariate Models

The next stage in our analysis was to develop multivariate models with covariates on the data and study levels (see Table 2). The fit of the models was improved by the inclusion of 3 sets of factors that varied within and between studies: population characteristics, the choice of intervention and comparators, and certain methods of analysis. In comparison with the variance components models (Table 1), the DIC statistics were lower for all 3 multivariate models.

**Table 2:** Multivariate Models with Covariates on Data and Study Levels

			Hierarchical Model		Hierarchical Hybrid Model		Cross-Classified Model	
		Raw Mean (SD) / n(%)	ΔC (SE)	ΔE (SE)	ΔC (SE)	ΔE (SE)	ΔC (SE)	ΔE (SE)
	Intercept	ΔC 8872 (15062) ΔE 0.636 (0.908)	15,297 (2592)	0.501 (0.162)	14,399 (2396)	0.584 (0.159)	14,459 (2073)	0.567 (0.13)
Patient and disease	TC	6.676 (1.204)	−3234 (350)	0.059 (0.017)	−3219 (333)	0.037 (0.019)	−3283 (329)	0.034 (0.02)
	HDL	1.168 (0.102)	11733 (6024)	−1.149 (0.328)	11222 (5788)	−0.581 (0.328)	9347 (5522)	−0.64 (0.34)
	SBP	137.48 (13.348)	−336 (22)	0.012 (0.001)	−330 (20.9)	0.012 (0.001)	−328 (20.8)	0.01 (0.001)
	Diabetes	17.81 (34.91)	−5284 (1561)	0.686 (0.077)	−1737 (972)	0.405 (0.055)	−2282 (895)	0.400 (0.055)
	Age (years)							
	<45	322 (15.38%)	Reference	Reference	Reference	Reference	Reference	Reference
	46–55	439 (20.96%)	−9346 (721)	−0.264 (0.034)	−9551 (647)	−0.313 (0.036)	−9565 (650)	−0.315 (0.036)
	56–65	862 (41.17%)	−12962 (704)	−0.527 (0.034)	−13454 (633)	−0.641 (0.035)	−13522 (641)	−0.644 (0.036)
	66–75	299 (14.28%)	−16353 (755)	−0.856 (0.036)	−16854 (676)	−1.070 (0.038)	−16922 (672)	−1.071 (0.037)
	>75	98 (4.68%)	−12765 (1220)	−0.560 (0.058)	−13107 (1139)	−0.651 (0.064)	−13187 (1141)	−0.657 (0.063)
	unclear	74 (3.53%)	−9903 (4589)	−0.190 (0.254)	−10387 (4525)	−0.253 (0.273)	−9911 (4369)	−0.203 (0.287)
	Gender							
	Female	576 (27.51%)	Reference	Reference	Reference	Reference	Reference	Reference
	Male	799 (38.16%)	−3647 (547)	0.129 (0.027)	−3539 (486)	0227 (0.027)	−3612 (485)	0.336 (0.027)
	Mixed sample	719 (34.34%)	−4224 (2558)	0.041 (0.150)	−3752 (2163)	0.108 (0.137)	−2940 (2298)	0.149 (0.138)
	CVD history							
	No	1064 (50.81%)		Reference		Reference		Reference
	Yes	958 (47.75%		0.209 (0.051)		0.178 (0.051)		0.161 (0.051)
	Mixed sample	72 (3.44%)		0.243 (0.231)		0.309 (0.253)		0.256 (0.239)
Intervention/comparator	cost_int.	528.84 (326.32)	11.9 (1.1)		13.1 (1.1)		12.8 (1.1)	
	cost_comp.	26.09 (115.31)	−13.4 (2.6)		−14.5 (2.4)		−14.2 (2.3)	
	active_comp.							
	Doing nothing	1834 (87.58%)		Reference		Reference		Reference
	Other statin	260 (12.42%)		−0.299 (0.072)		−0.298 (0.078)		−0.302 (0.076)
Methods	DRC	3.9 (1.7)	665 (163)		726 (155)		686 (154)	
	DRB	0.030 (0.018)				−0.05 (0.01)		−0.049 (0.009)
	Time horizon							
	<20 y	670 (32.00%)		Reference		Reference		Reference
	Lifetime	1424 (68.00%)		0.131 (0.041)		0.138 (0.044)		0.139 (0.045)
	Base case							
	Yes	1125 (53.72%)	Reference		Reference		Reference	
	No	969 (46.28%)	2941 (576)		2867 (503)		2750 (5031)	
	Effect calculation							
	CVD-red.	793 (37.87%)	Reference	Reference	Reference	Reference	Reference	Reference
	Chol-red.	1301 (62.13%)	6302 (2906)	0.334 (0.174)	7365 (2492)	0.351 (0.175)	8267 (2530)	0.423 (0.172)
Random part	$\sqrt{\sigma_{{u0}_{j}}^{2}}$(Country)		5397	0.330	4674	0.287	1099	0.053
	$\sqrt{\sigma_{{u0}_{k}}^{2}}$(Study)		8400	0.547	8003	0.550	8657	0.581
	$\sqrt{\sigma_{c0}^{2}}$(Data)		8511	0.403	8002	0.445	7999	0.445
	Country VPC (%)		16.92	19.03	14.57	14.17	0.86	0.52
	Study VPC (%)		41.00	52.46	42.72	51.91	53.48	62.66
	Data VPC (%)		42.08	28.51	42.71	33.92	45.66	36.82
	DIC		22,908		26,749		26,749	

Note: CVD = cardiovascular disease; ΔC = incremental cost; ΔE = incremental effect; DIC = deviance information criterion; DRB = discount rate (benefits); DRC = discount rate (cost); HDL = high-density lipoprotein; SBP = systolic blood pressure; SD = standard deviation; SE = standard error; TC = total cholesterol; VPC = variance partitioning coefficient. Covariate definitions are also provided in Supplementary Appendix A.

The direction and magnitude of the estimated coefficients were similar across the model specifications. Indicators of higher baseline CVD risk were associated with higher ΔE and lower ΔC: higher total cholesterol, lower high-density lipoprotein, higher systolic blood pressure, diabetes, and male gender. This fits expectations, as statins have the potential to prevent CVD events and hence to produce gains in (quality-adjusted) life-years and reductions in CVD-related expenditure in patients at higher risk. Older age was associated with lower ΔE and ΔC. This may seem counterintuitive, as older people are at greater risk of CVD. However, older people also have fewer years of life/QALYs to be gained from treatment.^42^ CVD history was highly significant for ΔE, suggesting that secondary prevention patients tend to benefit more from statin therapy. As might be expected, ΔC were positively related to intervention cost and negatively related to comparator cost. The binary variable “active comparator” was also statistically significant, indicating that comparisons against another statin tended to have lower ΔE and ΔC than comparisons against no treatment or placebo.

Regarding methodological factors, discount rates had highly significant coefficients that were positive for ΔC and negative for ΔE. This may be understood by considering the likely distributions of costs and effects over time. As a cohort ages, their CVD risk rises, and so the total number of events avoided and health gains from prevention by statins will also rise. Thus, a higher discount rate results in lower ΔE. However, the cost of statin therapy is increasingly offset by savings in CVD treatment over time. A higher discount rate may therefore reduce ΔC if the reduction in the net present value of savings outweighs that of the costs. Not surprisingly, ΔE was higher with a longer time horizon (20 y or more). We also found that studies modeling the effects of statins indirectly via their effect on cholesterol levels tended to show higher ΔC and ΔE than studies that estimated the effect on CVD outcomes directly. Finally, we found that results from base case analyses tended to have lower incremental cost compared with results from sensitivity analyses, potentially indicating a bias in the reporting of sensitivity analyses in published papers.

Running models with multiple covariates on data and study levels changed the percentage of variability found on country level for the fully hierarchical and the hierarchical hybrid models. The country VPC remained negligible below 1% for both ΔC and ΔE in the cross-classified model. These changes in country VPC accord with MLM theory, as the inclusion of lower-level covariates may increase, reduce, or leave unchanged the variability found on higher levels.^29,37,38^

We then moved on to explore whether between-country variation was associated with any measureable national characteristics. However, as the number of countries in the data set was relatively low, we tested only 1 country covariate at a time, adding each to the previously specified models with covariates included on data and study level (Table 2). The estimated means and standard errors for the country-level covariates, and the changes in DIC statistics, are shown in Table 3.

**Table 3:** Country-Level Covariates Tested in the Multivariate Models

Covariate		Raw Mean (SD)	Hierarchical Model		Hierarchical Hybrid Model		Cross-Classified Model	
			ΔC (SE)	ΔE (SE)	ΔC (SE)	ΔE (SE)	ΔC (SE)	ΔE (SE)
HC finance characteristics	GDP	35,169 (8969)	−0.28*** (0.09)	0.00 (0.00)	−0.21*** (0.08)	0.00 (0.00)	0.06 (0.1)	0.00 (0.00)
			DIC: –20		DIC: –10		DIC: +1	
	THE_GDP	9.96% (2.38)	−402.4*** (149.9)	0.021** (0.01)	−321.4** (151.7)	0.02** (0.01)	420.9 (298.7)	0.006 (0.017)
			DIC: –11		DIC: –7		DIC: –1	
	GOV_EXP_THE	68.18% (11.44)	64.3 ** (28.9)	0.002 (0.002)	58.7** (29.3)	0.001 (0.002)	−50.7 (62.7 )	−0.001 (0.004)
			DIC: –4		DIC: –1		DIC: –1	
	PRIV_EXP_THE	29.67% (12.85)	−61.1** (28.8)	0.002 (0.002)	−55.8* (29.7)	0.000 (0.002)	59.3 (58.9)	0.001 (0.003)
			DIC: –4		DIC: –2		DIC: –1	
HC ressource availability	GPs/10.000	29.13 (7.49)	−52.8 (143.9)	0.002 (0.007)	20.8 (97.7)	0.008 (0.005)	25.6 (88.8)	−0.001 (0.005)
			DIC: +2		DIC: –1		DIC: +2	
	NURSES/10.000	82.03 (45.27)	14.3 (15.4)	0.001 (0.001)	10.9 (13.8)	0.001 (0.001)	0.52 (15.7)	−0.000 (0.001)
			DIC: +1		DIC: +1		DIC: +1	
	PHARMACISTS/10.000	7.74 (3.24)	−12.9 (237.9)	0.032*** (0.011)	−101.7 (199.8)	0.021** (0.011)	−270.7 (228.5)	0.009 (0.013)
			DIC: –7		DIC: –3		DIC: –1	
	BEDS/10.000	51.37 (28.88)	32.6 (48.0)	0.001 (0.002)	19.9 (26.8)	0.002 (0.002)	−17.8 (27.3)	0.000 (0.002)
			DIC: +2		DIC: –1		DIC: +1	
Population demographic and disease characteristics	AGE/population	39.87 (3.50)	415.7* (227.7)	0.01 (0.013)	376.5* (200.0)	0.022** (0.011)	−73.1 (264.4)	−0.002 (0.015)
			DIC: –1		DIC: –5		DIC: +1	
	LIFE_EXPECTANCY at birth	80.06 (2.80)	101.6 (562.4)	0.039 (0.028)	3.0 (365.9)	0.012 (0.021)	−427.1 (469.2)	0.015 (0.028)
			DIC: +0		DIC: +1		DIC: +0	
	MEAN_BMI/Population	26.52 (1.22)	−1718** (776)	0.110** (0.045)	−537.9 (580.7)	0.05 (0.033)	911.1 (718.4)	0.013 (0.041)
			DIC: –11		DIC: –1		DIC: +0	
	MEAN_GLUCOSE/ Population	4.49 (0.17)	−4482 (3010)	0.379** (0.163)	−2958 (2797)	0.356** (0.153)	1596 (4231)	0.121 (0.246)
			DIC: –6		DIC: –5		DIC: +1	

Note: BMI = body mass index; ΔC = incremental cost; ΔE = incremental effect; DIC = deviance information criterion; GDP = gross domestic product; GPs = general practitioners; SD = standard deviation; SE = standard error; THE = total health expenditure; VPC = variance partitioning coefficient. Covariate definitions are also provided in Supplementary Appendix A.

*Significant at the 10% level. **Significant at the 5% level. ***Significant at the 1% level.

With respect to ΔC, we found highly significant negative coefficients for both GDP per capita and the percentage of GDP spent on health care in the hierarchical and hybrid models. This accords with Grieve and others,^21^ who assessed multicenter observational data of the cost and outcomes of stroke care and found that, for centers in countries that spent a lower percentage of GDP on health care, the intervention was not cost-effective, whereas it was cost-saving for countries with medium or high percentages of GDP spent on health care. A potential explanation for this is that countries that spend a higher proportion of GDP on health care may have higher average patient cost, which implies higher potential cost savings from CVD prevention.^20,22^ We also found highly significant positive coefficients for government spending as a percentage of total health care spending. This may be due to more rigorous cost-containment policies in countries in which health care is predominantly publicly funded, resulting in lower average cost and less potential for future cost-savings from statin prevention. We further found positive and significant coefficients for population age in both the hierarchical and hybrid model, indicating that incremental costs of statin prevention are higher in older populations.

With respect to ΔE, we found only small but positive and significant coefficients for the percentage of GDP spent on health care, the number of pharmacists per 10,000 population, as well as mean population age, body mass index, and glucose levels in either the hierarchical or the hybrid model specifications. The positive relationship with population-level risk factors fully accords with prior expectations as statins prevention should be more effective in higher-risk groups. The same holds for the number of pharmacists per 10,000 population, which was the only resource-related covariate on country level for which we found significant coefficients within the statins case study. In general, however, variation in ΔE between countries was more difficult to explain, which may be due to the tendency to assume generalizability of clinical data when populating economic models.^6^

## Discussion

Some commentators have argued that economic evaluation results are not transferable: “The economic question of whether an activity adds more to well-being than the alternative use of the same resources in a particular community cannot be answered by reference to the costs and consequences of the same activity in a different community.”^43(p218)^ Others have argued that transfer might sometimes be appropriate and suggested criteria for when this might be the case (e.g., refs. 44–46). We developed methods to assess the extent of variation in cost-effectiveness estimates that is attributable to differences between countries and to investigate whether this country-level variation can be explained by measureable factors. This method could potentially be used to provide empirical evidence to support or refute contentions about the appropriateness of transferring cost-effectiveness estimates from one context to another. We applied our method to a case study of statins for the prevention of cardiovascular disease.

### Summary of Key Findings

One of the most important, and unexpected, findings of our analysis was the relatively low proportion of variation attributable to the country level as compared with that at the data and study levels. The country-level VPC differed between model specifications but never exceeded 17% for ΔC or 21% for ΔE. This does not mean that differences between countries do not matter in economic evaluations but rather that they may not be fully reflected in published cost-effectiveness data. Barbieri and others^25^ explored issues surrounding variability of economic evaluations of pharmaceuticals in Western Europe and found considerable variation between countries. However, they did not attempt to assign variance to differences in study questions or methods, so all observed variability was attributed to differences between countries.

Looking at the extent of intercountry variation estimated from multinational studies, we found that by pooling multinational data in a separate group in the hierarchical hybrid model, estimates of country-level variability increased as compared with the cross-classified specification. Our finding accords with Barbieri and others’^25(p15)^ observation that “the extent of variability is lower for multicountry studies than single country studies” and “this may be because, in a multicountry study, the analysts give more active consideration to the harmonization of data and analyses.” This does, however, leave us with the question of whether the multinational studies are underestimating true variation between countries or whether single-country studies are overestimating it. Given this uncertainty, it is difficult to conclude whether pooling of multinational and single-study data in their respective country categories as in the cross-classified model is appropriate.

### Advantages of Our Approach

The multilevel approach allowed us to separate effects of differences within studies, between studies, and between countries. Using models that recognized the hierarchical structure of the data, adjustment for confounding effects and the use of a bivariate response vector of correlated incremental costs and effects changed the estimated proportion of variation attributable to countries. Further improvements to our model structure or covariates might possibly further change these results. We cannot claim that the final models presented are the best of all possible models, but they did offer the best fit across a large number of specifications tested. The estimated coefficients are generally plausible in size and direction, and they were robust to model specification and the inclusion of other covariates and across subsets of data. A further strength of our analysis is that the explanatory variables were derived systematically from a list of factors previously suggested in the literature.^6,7^

### Study Limitations

An important limitation of our analysis is that it was based on a single case study. The topic of statins was chosen as it has been extensively researched. However, all countries in the data set are developed countries, with similar levels of economic and health indicators. The generalizability of our findings to other clinical topics and countries is uncertain.

Our model is predicated on the assumption that the reported results of published cost-effectiveness studies are exchangeable. This may be challenged because of potential relationships between studies, for instance, through common authorship, data sources, or models. As an example of the latter, we found that estimates based on the CVD-life expectancy model from different studies were highly dependent.^47^ The relative similarity of estimates across countries might therefore say more about the willingness of health economists to borrow from one another than about true commonality of cost-effectiveness in different contexts. The assumption of random parameters on study and country level may also be violated as we excluded studies that did not report both ΔC and ΔE. Earlier studies may have been more likely to report results as incremental cost-effectiveness ratios (ICERs; ΔC/ΔE), which may have biased the data set toward more recent studies. This might possibly have affected the variability observed between studies, as more recent studies may build on experiences (and results) of earlier studies, which may lead to a converging effect in terms of variability over time.

There is also good reason to suspect cross-level correlation between random effects on study and country level. For instance, if national health technology assessment guidelines govern the choice of methods, some country-level variability may be dragged down to the study level. However, the timing of studies ranged from 1988 to 2009, a period that started before methods guidelines were established and during which existing guidelines repeatedly changed. In addition, some authors may have targeted an international rather than a national audience, trying to balance different national methods guidelines. Accordingly, we could not find a correspondence between methods and countries, using multilevel analyses or with multiple correspondence analyses.^48^

Another potential objection to our approach relates to the use of multiple cost-effectiveness estimates from each published study. This adds power, as sensitivity and subgroup analyses provide information about variation in ΔE and ΔC as a function of different specifications of the problem (differences in population, intervention, and comparator) and methodological assumptions (e.g., discount rate or costing perspective). However, it might appear problematic that studies that report a larger number of analyses receive more weight in our model. In the MLM framework, study and country group means are estimated by weighting mean raw residuals according to the number of ΔC and ΔE estimates in each group, as well as the variances of the within- and between-group error terms.^11,29,37,38^ We tested the impact of group sizes on group means and found it to be small or modest for most studies included in the analysis.^49^

A more usual approach in meta-regression is to weight studies according to the precision of their results. However, estimates of precision are not available for a large majority of cost-effectiveness studies. This is likely to be a general characteristic of the cost-effectiveness literature, rather than a particular flaw in our data set. Trial-based evaluations might report the variance for ΔC and ΔE, although they rarely report a measure of covariance between these outcomes. More importantly, there is a fundamental problem in obtaining estimates of precision from model-based economic evaluations. Uncertainty in decision models is not just a function of sampling variation for the effectiveness data; it is also related to uncertainty over a range of other input parameters and assumptions used to extrapolate trial results or to estimate associated costs and QALYs. These uncertainties are explored using various forms of sensitivity analysis and reported in various ways, for example, as confidence intervals around an ICER or in a cost-effectiveness acceptability curve estimated from a probabilistic sensitivity analysis (PSA). The closest analogue of precision for a modeling study would be the covariance matrix for ΔC and ΔE resulting from a PSA. However, this is rarely reported directly, and furthermore, it is susceptible to bias due to discretion over which input parameters are included in the PSA and how uncertainty is reflected in the form of probability distributions for those inputs. Thus, estimates of precision from decision analytic models, when available, are rarely compatible. We could have excluded such model-based estimates from our analysis; however, this would not only have severely limited the size of our sample but, more importantly, it would also have provided an unrepresentative sample from the population of published cost-effectiveness results available to inform policy decisions and compromised our ability to investigate the transferability of that evidence base.

### Areas for Further Research

A key remaining question is whether it is possible to use our models to predict cost-effectiveness in countries for which information is currently lacking. The reliability of any such predictions would rest on identification of the appropriate set of covariates for the exchangeability assumption to hold, and representation of the characteristics of the country of interest by the countries in the dataset.^27^ However, more research is needed to establish the appropriate covariates and to test whether they are robust across a wider range of countries. The contribution of this article has been to suggest methods to start to investigate these questions, but we believe it would be premature to use our results to decide whether or not to transfer or adapt results to a particular context.

The key priority for further research is to replicate our approach for other clinical topics, particularly ones for which a wider range of country estimates might be available. We are also keen to further investigate the genealogy of economic evaluations and its effects on variation in cost-effectiveness estimates. Finally, further research into the development of MLM techniques for meta-regression of cost-effectiveness information is required.

## Conclusion

Our analysis for the statins case study suggests that variability in published cost-effectiveness data is primarily due to differences between studies, not countries. Nevertheless, decision makers ought to be critical before using data from other contexts because of the additional uncertainty attached to the transferred evidence. What remains problematic is how to find the appropriate set of covariates for the exchangeability assumption to hold. We have set out a possible method to provide an empirical answer to this question, but further work is needed to test the validity of this approach and to investigate the robustness of conclusions about the extent of variations in cost-effectiveness between countries and whether common factors exist that are predictive of such differences.

## Supplementary Material

Supplementary material

## Supplementary Material

Supplementary material

## Supplementary Material

Supplementary material
