# Supplementary material for: Mind the Gap! A Multilevel Analysis of Factors Related to Variation in Published Cost-Effectiveness Estimates within and between Countries
Source: Med Decis Making. 2016 Jan;36(1):31–47. doi: 10.1177/0272989X15579173 (PMC4708620; doi:10.1177/0272989X15579173)
Supplement: Supplementary material [file DS_10.11770272989X15579173_Appendix_A.pdf]

Appendix A. Covariates for multilevel analysis on data, study and country-level

Variable name	Description	Level	Nature of variable
	Group A: patient and disease characteristics*		
AGE_CAT	*Age of patient subgroup*	*Level 1 (data)*	*ordered, categorical*
GENDER	*Gender of patient subgroup*	*Level 1 (data)*	*unordered, categorical*
CVD_HISTORY	*Cardiovascular Disease (CVD) related medical history of patient subgroup*	*Level 1 (data)*	*unordered, categorical*
TC	*Total Cholesterol (TC) at baseline*	*Level 1 (data)*	*Continuous*
HDL	*High Density Lipoprotein (HDL) at baseline*	*Level 1 (data)*	*Continuous*
LDL	*Low Density Lipoprotein (LDL) at baseline*	*Level 1 (data)*	*Continuous*
HYPERTENSION	*Percentage of hypertensive patients in subgroup*	*Level 1 (data)*	*Continuous*
SBP	*Mean Systolic Blood Pressure (SBP) at baseline*	*Level 1 (data)*	*Continuous*
DIABETES	*Percentage of diabetic patients at baseline*	*Level 1 (data)*	*Continuous*
SMOKERS	*Percentage of smokers at baseline*	*Level 1 (data)*	*Continuous*
BP_PCF	*Principal components factor score of 'SBP', 'HYPERTENION' and 'SMOKERS', representing circulation related CVD risk*	*Level 1 (data)*	*Continuous*
RISK_CAT	*CVD risk of patient subgroup at baseline (estimated from patient subgroup characteristics using Framingham risk equation)*	*Level 1 (data)*	*ordered, categorical*
	Group B intervention and comparator characteristics*		
INTERVENTION	*Brand name of intervention*	*Level 1 (data)*	*unordered, categorical*
COMPARATOR	*Brand name of comparator*	*Level 1 (data)*	*unordered, categorical*
ACTIVE_COMP	*Yes if intervention was compared to other statin and/or different dosage*	*Level 1 (data)*	*unordered, binary*
TDD_INT	*Total Daily Dose (TDD) of intervention*	*Level 1 (data)*	*ordered, categorical*
TDD_COMP	*Total Daily Dose (TDD) of comparator*	*Level 1 (data)*	*ordered, categorical*
COST_INT	*Annual drug cost of intervention in 2010 £-Sterling*	*Level 1 (data)*	*Continuous*
UNITCOST_INT	*Unit cost of intervention (£-Sterling / mg)*	*Level 1 (data)*	*Continuous*
COST_COMP	*Annual drug cost of comparator in 2010 £-Sterling*	*Level 1 (data)*	*Continuous*
UNITCOST_COMP	*Unit cost of comparator (£-Sterling / mg)*	*Level 1 (data)*	*Continuous*
INCR_DRUG_COST	*Incremental annual drug cost of intervention versus coparator*	*Level 1 (data)*	*Continuous*
	Group C: methodological characteristics on data-level*		
OUTC_MEASURE	*Life Year Saved (LYS) or Quality Adjusted Life Year (QALYs)*	*Level 1 (data)*	*unordered, binary*
ELICITATION	*If QALYS, what was the method of preference elicitation?*	*Level 1 (data)*	*unordered, categorical*
POPULATION	*If QALYS, were utility values elicited from patients or general population?*	*Level 1 (data)*	*unordered, categorical*
DRC	*Discount rate for costs (DRC)*	*Level 1 (data)*	*Continuous*
DRB	*Discount rate for benefits (DRB)*	*Level 1 (data)*	*Continuous*
DURATION	*Treatment duration*	*Level 1 (data)*	*ordered, categorical*
EXTRAPOLATION	*Was there any extrapolation beyond the latest follow up?*	*Level 1 (data)*	*unordered, binary*
HORIZON	*What was the time horizon of the analysis?*	*Level 1 (data)*	*ordered, categorical*
DURATION=HORIZON	*Does the time horizon equal the treatment duration?*	*Level 1 (data)*	*unordered, binary*
PERSP_REP	*What was the study perspective as reported by the authors of the article?*	*Level 1 (data)*	*unordered, categorical*
PERSP_COST_CONCL	*What was the study perspective on costs as concluded by the reviewer?*	*Level 1 (data)*	*unordered, categorical*
PERSP_BEN_CONCL	*What was the study perspective on effects as concluded by the reviewer?*	*Level 1 (data)*	*unordered, categorical*
DATA_CLASS	*Estimate from base case or different categories of sensitivity analyses?*	*Level 1 (data)*	*unordered, categorical*
BASECASE	*Base case analysis (yes / no)?*	*Level 1 (data)*	*unordered, binary*
SOURCE_EFFECTS	*From which source (trial, meta-analysis) was effectiveness data taken?*	*Level 1 (data)*	*unordered, categorical*
CONTEXT_ SPECIFICITY	*Variable which encodes the context specificity of input parameters. (four categories ranging from 'only one group of input parameters context specific' up to 'all groups of input parameters context specific') – adapected from Barbieri et al. (2005)*	*Level 1 (data)*	*ordered, categorical*
	Group D: general study characteristics*		
LANGUAGE	*In which language was the paper written?*	*Level 2 (study)*	*unordered, binary*
PAPER_ORIGIN	*From which country does the paper originate? (not necessarily identical to target country) If authors from several jurisdictions were involved, where was the lead author based?*	*Level 2 (study)*	*unordered, categorical*
TIMING	*What is the timing of the economic evaluation?*	*Level 2 (study)*	*unordered, categorical*
FUND_INST	*What was the primary source of funding for the study (institution)?*	*Level 2 (study)*	*unordered, categorical*
FUND_MAN	*If the funding source was private, which manufacturer was the primary sponsor of the study?*	*Level 2 (study)*	*unordered, categorical*
AUTHOR_GROUP	*Variable which encodes relationships between published papers in terms of common authorship*	*Level 2 (study)*	*unordered, categorical*
CVD_LIVE_ EXP_MODEL	*Variable which encodes whether study was based on the CVD life-expectancy model by Grover et al. (1998)*	*Level 2 (study)*	*unordered, binary*
	Group E: methodological characteristics on study-level*		
GEN_DES	*What was the general study design?*	*Level 2 (study)*	*unordered, categorical*
PRIM_DES	*If primary modelling, what was the specific study design?*	*Level 2 (study)*	*unordered, categorical*
SEC_DES	*If secondary modelling, what was the specific study design?*	*Level 2 (study)*	*unordered, categorical*

EFFECT_CALC	*Method of effect calculation: statin effectiveness was either estimated directly as reduction in CVD-risk (CVD-red.) or indirectly via a reduction in cholesterol levels which leads to a reduction in CVD-risk (chol-red.)*	*Level 2 (study)*	*unordered, binary*
MULTINATIONAL	*Was the study multinational?*	*Level 2 (study)*	*unordered, binary*
INFL_ADJ	*Were cost estimates in the model adjusted for inflation?*	*Level 2 (study)*	*unordered, categorical*
ADJ_METHOD	*If cost estimates were adjusted for inflation, which adjustment method was used?*	*Level 2 (study)*	*unordered, categorical*
CURRENCY_CONV	*Were currencies converted?*	*Level 2 (study)*	*unordered, binary*
CONV_METHOD	*If currencies were converted, which conversion method was used?*	*Level 2 (study)*	*unordered, categorical*
SCOPE	*What was the scope of assessment (i.e. Coronary Heart Diseas (CHD); CHD and stroke; or CHD, stroke and Peripherial Arterial Disease (PAD))*	*Level 2 (study)*	*unordered, categorical*
Group F: study quality indicators*			
QHES_CONT_A	*Quality of Health Economic Studies (QHES) score (strict application of QHES criteria, i.e. if a QHES dimension is only partly satisfied, no points are assigned to that dimension)*	*Level 2 (study)*	*continuous*
QHES_CONT_B	*Quality of Health Economic Studies (QHES) score (pragmatic application of QHES criteria, i.e. if a QHES dimension is only partly satisfied, a partial score is assigned to that dimension)*	*Level 2 (study)*	*continuous*
QHES_CAT_A	*QHES-score_a transformed into a categorical variable with 5 categories ranging from zero to 100*	*Level 2 (study)*	*ordered, categorical*
QHES_CAT_B	*QHES-score_b transformed into a categorical variable with 5 categories ranging from zero to 100*	*Level 2 (study)*	*ordered, categorical*
Group G: healthcare finance characteristics†			
GDP	*Gross Domestic Product (GDP) per capita in current international $, 2009*	*Level 3 (country)*	*Continuous*
THE_GDP	*Total health expenditure (THE) as percentage of GDP, 2009*	*Level 3 (country)*	*Continuous*
GOV_EXP_THE	*General government expenditure on health as percentage of total health expenditure, 2009*	*Level 3 (country)*	*Continuous*
PRIV_EXP_THE	*Private expenditure on health as percentage of total health expenditure, 2009*	*Level 3 (country)*	*Continuous*
Group H: healthcare resource availability			
GPS	*Physicians density (per 10 000 population) 2005-2009*	*Level 3 (country)*	*Continuous*
NURSES	*Nursing and midwifery personnel density (per 10 000 population) 2005-2009*	*Level 3 (country)*	*Continuous*
PHARMACISTS	*Density of pharmaceutical personnel (per 10 000 population) 2005-2009*	*Level 3 (country)*	*Continuous*
BEDS	*Hospital beds per (10 000 population) 2008 – 2010*	*Level 3 (country)*	*Continuous*
Group I: population demographic & disease characteristics†			
AGE_POPULATION	*Population median age (years), 2009*	*Level 3 (country)*	*Continuous*
LIFE_EXPECTANCY	*Life expectancy at birth (in years), 2009*	*Level 3 (country)*	*Continuous*
MEAN_BMI	*Mean Body Mass Index (BMI), crude estimate, 2008*	*Level 3 (country)*	*Continuous*
MEAN_GLUCOSE	*Mean fasting glucose in mmol/L, crude estimate, 2008*	*Level 3 (country)*	*Continuous*

* Sources for data and study-level covariates: Individual studies included in systematic literature review

† Sources for country-level covariates: WHO (2012) / World Bank (2012)
