# Supplementary material for: Mind the Gap! A Multilevel Analysis of Factors Related to Variation in Published Cost-Effectiveness Estimates within and between Countries
Source: Med Decis Making. 2016 Jan;36(1):31–47. doi: 10.1177/0272989X15579173 (PMC4708620; doi:10.1177/0272989X15579173)
Supplement: Supplementary material [file DS_10.11770272989X15579173_Appendix_B.pdf]

Appendix B. Key characteristics of included studies

Authors (publication year)	Timing	Multi-country	Target country	Primary Modelling	Outcome measure	Industry Funding
Alonso et al. (2008)	2005	No	Spain	No	LYS	Yes
Annemans et al. (2010)	2009	No	Belgium	No	LYS / QALYs	Yes
Ara et al. (2009)	2008	No	UK (England/Wales)	No	QALYs	No
Araujo et al. (2007)	2007	No	Brazil	No	LYS	Unclear
Ashraf et al. (1996)	1995	No	USA	No	LYS	Yes
Berger et al. (1997)	1996	No	Germany	No	LYS	Unclear
Caro et al. (1997)	1996	No	UK (Scotland)	Yes	LYS	Yes
Caro et al. (2003)	1998	No	USA	No	LYYS	Yes
CDC Group. (2002)	1997	No	USA	No	LYS / QALYs	No
Chan et al. (2007)	2005	No	USA	No	LYS / QALYs	No
Chau et al. (2001)	1998	No	Hong Kong	No	QALYs	Yes
Davies et al. (2006)	2005	No	UK	No	QALYs	Yes
Drummond et al. (1993)	1990	No	UK	No	LYS	No
Franco et al. (2007)	2003	No	Netherlands	No	LYS	No
Ganz et al. (2000)	1998	No	USA	No	LYS / QALYs	No
Glick et al. (1992)	1988	No	UK	No	LYS	Yes
Greving et al. (2011)	2008	No	Netherlands	No	QALYs	No
Grover et al. (1999)	1996	No	Canada	No	LYS	Unclear
Grover et al. (2000)	1996	No	Canada	No	LYS	Yes
Grover et al. (2001)	1998	Yes	Multi-country	No	LYS	Yes
Grover et al. (2003)	2000	No	Canada	No	LYS	Yes
Grover et al. (2008)	2002	No	Canada	No	LYS	Yes
Hamilton et al. (1995)	1992	No	Canada	No	LYS	Yes
Hjialte et al. (1989)	1988	No	Sweden	No	LYS	Unclear
HPS Group (2006)	2005	No	UK	No	LYS / QALYs	Yes
HPS Group (2009)	2006	No	USA	No	LYS / QALYs	Yes
Johanneson et al. (1997)	1995	No	Sweden	No	LYS	Yes
Johannesson et al (1996)	1991	No	Sweden	Yes	LYS	Yes
Jönsson et al. (1996)	1995	Yes	Multi-country	Yes	LYS	Yes
Jönsson et al. (1999)	1997	Yes	Multi-country	Yes	LYS	Yes
Khoury et al. (2009)	2007	No	Canada	No	LYS / QALYs	Yes
Kongnakorn et al. (2009)	2005	No	USA	No	LYS / QALYs	Yes
Lindgren et al. (2007)	2005	Yes	Multi-country	No	LYS / QALYs	Yes
Lindgren et al. (2010)	2007	No	UK (England/Wales)	No	LYS / QALYs	Yes
Martens et al. (1994)	1993	No	Canada	No	LYS	Yes
Morris & Godber (1999)	1997	No	Canada	No	LYS	Yes
Morris (1997)	1996	No	UK	No	LYS	Unclear
Muls et al. (1998)	1995	Yes	Belgium	No	LYS	Yes
Nagata et al. (2005)	2002	No	Japan	No	QALYs	Unclear
Nherera et al. (2010)	2009	No	UK (England/Wales)	No	QALYs	Unclear
NICE (2008)	2007	No	UK (England/Wales)	No	QALYs	No
Obermann et al. (1997)	1993	No	Germany	No	LYS	Yes
Perreault et al. (1998)	1995	No	Canada	No	LYS	No
Peura et al. (2008)	2006	No	Finland	No	LYS / QALYs	Yes
Pharoah et al. (1996)	1995	No	UK (England/Wales)	No	LYS	No
Raikou et al. (2007)	2004	No	UK	Yes	LYS / QALYs	Yes
Ramsey et al. (2008)	2005	No	USA	No	LYS / QALYs	Yes
Rosen (2010)	2007	No	USA	No	LYS / QALYs	Yes
Scuffham et al. (2004)	2002	No	UK (England/Wales)	No	LYS / QALYs	Yes
Scuffham et al. (2005)	2002	No	UK	No	LYS / QALYs	Yes
Scuffham et al. (2006)	2005	No	Hungary	No	LYS / QALYs	Yes
Sigvant et al. (2011)	2009	No	Sweden	No	LYS / QALYs	Yes
Slejko et al. (2010)	2008	No	USA	No	QALYs	Unclear
Soini et al. (2010)	2007	No	Finland	No	LYS / QALYs	Yes
Spaans et al. (2003)	1996	No	Canada	No	LYS	Unclear
Szucs et al. (1998)	1996	No	Germany	No	LYS	Unclear
Szucs et al. (2000a)	1998	No	Germany	No	LYS	Unclear
Szucs et al. (2000b)	1997	No	Switzerland	No	LYS	Unclear
Szucs et al. (2004)	2003	Yes	Multi-country	No	LYS	Unclear
Tailor et al. (2009)	2005	Yes	Multi-country	No	LYS / QALYs	Unclear
Tonkin et al. (2006)	1998	No	Australia	Yes	LYS	Yes
Troche et al. (1998)	1995	No	Germany	No	LYS	Unclear
Tsevat et al. (2001)	1996	No	USA	No	QALYs	Yes
van Hout et al. (2001)	1999	No	Netherlands	No	LYS	Unclear
Wagner et al. (2009a)	2007	No	Canada	No	LYS / QALYs	Yes
Wagner et al. (2009b)	2006	No	Canada	No	LYS / QALYs	Yes
Ward et al. (2007)	2004	No	UK (England/Wales)	No	QALYs	No
Abbreviations: LYS: Life Year Saved; QALY: Quality Adjusted Life Year						
