# Supplementary material for: Mind the Gap! A Multilevel Analysis of Factors Related to Variation in Published Cost-Effectiveness Estimates within and between Countries
Source: Med Decis Making. 2016 Jan;36(1):31–47. doi: 10.1177/0272989X15579173 (PMC4708620; doi:10.1177/0272989X15579173)
Supplement: Supplementary material [file DS_10.11770272989X15579173_Appendix_C.pdf]

Appendix C. References of included studies

ALONSO, R., FERNANDEZ DE BOBADILLA, J., MENDEZ, I., LAZARO, P., MATA, N. & MATA, P., 2008. Cost-effectiveness of managing familial hypercholesterolemia using atorvastatin-based preventive therapy. *Rev. Esp. Cardiol.*, 61(4), pp. 382-393.

ANNEMANS, L., MARBAIX, S., WEBB, K., VAN GAAL, L. & SCHEEN, A., 2010. Cost effectiveness of atorvastatin in patients with type 2 diabetes mellitus - a pharmacoeconomic analysis of the collaborative atorvastatin diabetes study in the Belgian population. *Clinical Drug Investigation,* 30(2), pp. 133-142.

ARA, R., PANDOR, A., STEVENS, J., REES, A. and RAFIA, R., 2009. Early high-dose lipid-lowering therapy to avoid cardiac events: a systematic review and economic evaluation. *Health Technology Assessment,* 13(34), pp. 1-74.

ARAUJO, D.V., BAHIA, L., SOUZA, C.P.R. & PAVÃ£O, A.L.B., 2007. Cost-effectiveness and budget impact analysis of rosuvastatin and atorvastatin for LDL-cholesterol and cardiovascular events lowering within the SUS scenario. *International Journal of Atherosclerosis,* 2(3), pp. 189-194.

ASHRAF, T., HAY, J.W., PITT, B., WITTELS, E., CROUSE, J., DAVIDSON, M., FURBERG, C.D. & RADICAN, L., 1996. Cost-effectiveness of pravastatin in secondary prevention of coronary artery disease. *American Journal of Cardiology,* 78(4), pp. 409-414.

BERGER, K., KLOSE, G. & SZUCS, T.D., 1997. Economic aspects of drug therapy exemplified by pravastatin. A socioeconomic analysis of cholesterol synthase enzyme inhibition in coronary heart disease patients. *Medizinische Klinik,* 92(6), pp. 363-369.

CARO, J., KLITTICH, W., MCGUIRE, A., FORD, I., NORRIE, J., PETTITT, D., MCMURRAY, J. & SHEPHERD, J., 1997. The West of Scotland coronary prevention study: economic benefit analysis of primary prevention with pravastatin. *BMJ,* 315(7122), pp. 1577-1582.

CARO, J.J., HUYBRECHTS, K.F., KLITTICH, W.S., JACKSON, J.D. & MCGUIRE, A., 2003. Allocating funds for cardiovascular disease prevention in light of the NCEP ATP III guidelines. *American Journal of Managed Care,* 9(7), pp. 477-489.

CDC DIABETES COST-EFFECTIVENESS GROUP, 2002. Cost-effectiveness of intensive glycemic control, intensified hypertension control, and serum cholesterol level reduction for type 2 diabetes. *JAMA,* 287(19), pp. 2542-2551.

CHAN, P.S., NALLAMOTHU, B.K., GURM, H.S., HAYWARD, R.A. & VIJAN, S., 2007. Incremental benefit and cost-effectiveness of high-dose statin therapy in high-risk patients with coronary artery disease. *Circulation,* 115(18), pp. 2398-2409.

CHAU, J., CHEUNG, B.M., MCGHEE, S.M., LAUDER, I.J., LAU, C.P. & KUMANA, C.R., 2001. Cost-effectiveness analysis of applying the Cholesterol and Recurrent Events (CARE) study protocol in Hong Kong. *Hong Kong Medical Journal,* 7(4), pp. 360-368.

DAVIES, A., HUTTON, J., O'DONNELL, J. & KINGSLAKE, S., 2006. Cost-effectiveness of rosuvastatin, atorvastatin, simvastatin, pravastatin and fluvastatin for the primary prevention of CHD in the UK. *British Journal of Cardiology,* 13(3), pp. 196-202.

DRUMMOND, M.F., MCGUIRE, A. & FLETCHER, A., 1993. *Economic evaluation of drug therapy for hypercholesterolemia in the UK.* University of York, CHE discussion paper No 104.

FRANCO, O.H., DER KINDEREN, A.J., DE LAET, C., PEETERS, A. & BONNEUX, L., 2007. Primary prevention of cardiovascular disease: cost-effectiveness comparison. *International Journal of Technology Assessment in Health Care,* 23(1), pp. 71-79.

GANZ, D.A., KUNTZ, K.M., JACOBSON, G.A. & AVORN, J., 2000. Cost-effectiveness of 3-hydroxy-3-methylglutaryl coenzyme A reductase inhibitor therapy in older patients with myocardial infarction. *Annals of Internal Medicine,* 132(10), pp. 780-787.

GLICK, H., HEYSE, J.F., THOMPSON, D., EPSTEIN, R.S., SMITH, M.E. & OSTER, G., 1992. A model for evaluating the cost-effectiveness of cholesterol-lowering treatment. *International Journal of Technology Assessment in Health Care,* 8(4), pp. 719-734.

GREVING, J., VISSEREN, F., DE WIT, G. & ALGRA, A., 2011. Statin treatment for primary prevention of vascular disease: whom to treat? Cost-effectiveness analysis. *BMJ (Clinical research ed.),* 342, pp. d1672.

GROVER, S.A., COUPAL, L., PAQUET, S. & ZOWALL, H., 1999. Cost-effectiveness of 3-hydroxy-3-methylglutaryl-coenzyme A reductase inhibitors in the secondary prevention of cardiovascular disease: forecasting the incremental benefits of preventing coronary and cerebrovascular events. *Archives of Internal Medicine,* 159(6), pp. 593-600.

GROVER, S.A., COUPAL, L., ZOWALL, H. & DORAIS, M., 2000. Cost-effectiveness of treating hyperlipidemia in the presence of diabetes : who should be treated?. *Circulation,* 102(7), pp. 722-727.

GROVER, S.A., COUPAL, L., ZOWALL, H., ALEXANDER, C.M., WEISS, T.W. & GOMES, D.R.J., 2001. How Cost-Effective is the Treatment of Dyslipidemia in Patients with Diabetes but without Cardiovascular Disease? *Diabetes Care,* 24(1), pp.45-50

GROVER, S.A., HO, V., LAVOIE, F., COUPAL, L., ZOWALL, H. & PILOTE, L., 2003. The importance of indirect costs in primary cardiovascular disease prevention: Can we save lives and money with statins? *Archives of Internal Medicine,* 163(3), pp. 333-339.

GROVER, S.A., COUPAL, L. & LOWENSTEYN, I., 2008. Preventing cardiovascular disease among Canadians: Is the treatment of hypertension or dyslipidemia cost-effective? *Canadian Journal of Cardiology,* 24(12), pp.891-898

HAMILTON, V.H., RACICOT, F.E., ZOWALL, H., COUPAL, L. & GROVER, S.A., 1995. The cost-effectiveness of HMG-CoA reductase inhibitors to prevent coronary heart disease. Estimating the benefits of increasing HDL-C. *JAMA,* 273(13), pp. 1032-1038.

HEART PROTECTION STUDY COLLABORATIVE GROUP, 2009. Statin Cost-Effectiveness in the United States for People at Different Vascular Risk levels. *Circulation.Cardiovascular Quality & Outcomes,* 2(2), pp. 65-72.

HEART PROTECTION STUDY COLLABORATIVE GROUP, MIHAYLOVA, B., BRIGGS, A., ARMITAGE, J., PARISH, S., GRAY, A. and COLLINS, R., 2006. Lifetime cost effectiveness of simvastatin in a range of risk groups and age groups derived from a randomised trial of 20,536 people. *BMJ,* 333(7579), pp. 1145.
HJALTE, K., LINDGREN, B., PERSSON, U. & OLSSON, A.G., 1989. Lipid lowering therapy: Cost estimates in Sweden. In: Lewis, B., Assman, G., eds. *The social and economic context of coronary prevention. Current medical Literature*: Proceedings of the international task force for prevention of coronary heart disease.
JOHANNESSON, M., BORGQUIST, L., JONSSON, B. & LINDHOLM, L.H., 1996. The cost effectiveness of lipid lowering in Swedish primary health care. The CELL Study Group. *Journal of Internal Medicine,* 240(1), pp. 23-29.
JOHANNESSON, M., JONSSON, B., KJEKSHUS, J., OLSSON, A.G., PEDERSEN, T.R. & WEDEL, H., 1997. Cost effectiveness of simvastatin treatment to lower cholesterol levels in patients with coronary heart disease. *The New England Journal of Medicine,* 336, pp.332-336
JONSSON, B., COOK, J.R. & PEDERSEN, T.R., 1999. The cost-effectiveness of lipid lowering in patients with diabetes: results from the 4S trial. *Diabetologia,* 42(11), pp. 1293-1301.
JONSSON, B., JOHANNESSON, M., KJEKSHUS, J., OLSSON, A.G., PEDERSEN, T.R. & WEDEL, H., 1996. Cost-effectiveness of cholesterol lowering. Results from the Scandinavian Simvastatin Survival Study (4S). *European Heart Journal,* 17(7), pp. 1001-1007.
KHOURY, H., WAGNER, M., MERIKLE, E., JOHNSON, S.J. and ROBERTS, C., 2009. Cost-effectiveness of atorvastatin in the primary prevention of major cardiovascular events in patients with type 2 diabetes in Canada. *Canadian Journal of Diabetes,* 33(4), pp. 363-374.
KONGNAKORN, T., WARD, A., ROBERTS, C.S., O'BRIEN, J.A., PROSKOROVSKY, I. & CARO, J.J., 2009. Economic evaluation of atorvastatin for prevention of recurrent stroke based on the SPARCL trial. *Value in Health,* 12(6), pp. 880-887.
LINDGREN, P., ERIKSSON, J., BUXTON, M., KAHAN, T., POULTER, N., DAHLOF, B., SEVER, P., WEDEL, H., JONSSON, B. & ANGLO-SCANDINAVIAN-CARDIAC OUTCOMES TRIAL INVESTIGATORS, 2010. The economic consequences of non-adherence to lipid-lowering therapy: results from the Anglo-Scandinavian-Cardiac Outcomes Trial. *International Journal of Clinical Practice,* 64(9), pp. 1228-1234.
LINDGREN, P., GRAFF, J., OLSSON, A.G., PEDERSEN, T.J., JONSSON, B. & IDEAL TRIAL, I., 2007. Cost-effectiveness of high-dose atorvastatin compared with regular dose simvastatin. *European Heart Journal,* 28(12), pp. 1448-1453.
MARTENS, L.L. & GUIBERT, R., 1994. Cost-effectiveness analysis of lipid-modifying therapy in Canada: comparison of HMG-CoA reductase inhibitors in the primary prevention of coronary heart disease. *Clinical Therapeutics,* 16(6), pp. 1052-1062.
MORRIS, S., 1997. A comparison of economic modelling and clinical trials in the economic evaluation of cholesterol-modifying pharmacotherapy. *Health Economics,* 6(6), pp. 589-601.
MORRIS, S. & GODBER, E., 1999. Choice of cost-effectiveness measure in the economic evaluation of cholesterol-modifying pharmacotherapy. An illustrative example focusing on the primary prevention of coronary heart disease in Canada. *PharmacoEconomics,* 16(2), pp. 193-205.
MULS, E., VAN GANSE, E. & CLOSON, M.C., 1998. Cost-effectiveness of pravastatin in secondary prevention of coronary heart disease: comparison between Belgium and the United States of a projected risk model. *Atherosclerosis,* 137(Suppl), pp. S111-6.
NAGATA-KOBAYASHI, S., SHIMBO, T., MATSUI, K. & FUKUI, T., 2005. Cost-effectiveness of pravastatin for primary prevention of coronary artery disease in Japan. *International journal of cardiology,* 104(2), pp. 213-223.
NATIONAL INSTITUTE FOR HEALTH AND CLINICAL EXCELENCE (NICE), 2008 (reviewed 2010). *NICE clinical guideline 67 lipid modification, cardiovascular risk assessment and the modification of blood lipids for the primary and secondary prevention of cardiovascular disease; London, UK*
NHERERA, L., CALVERT, N.L., DEMOTT, K., HUMPHRIES, S.E., NEIL, H.A.W., MINHAS, R. & THOROGOOD, M., 2010. Cost-effectiveness analysis of the use of a high-intensity statin compared to a low-intensity statin in the management of patients with familial hypercholesterolaemia. *Current Medical Research and Opinion,* 26(3), pp.529-536
OBERMANN, K., MATTIAS, G., SCHULENBURG, J.M. & MAUTNER, G.C., 1997. Economic analysis of secondary prevention of coronary heart disease with simvastatin (Zocor) in Germany. *Medizinische Klinik,* 92(11), pp. 686-694.
PERREAULT, S., HAMILTON, V.H., LAVOIE, F. & GROVER, S., 1998. Treating hyperlipidemia for the primary prevention of coronary disease. Are higher dosages of lovastatin cost-effective? *Archives of Internal Medicine,* 158(4), pp. 375-381.
PEURA, P., MARTIKAINEN, P.P., SOINI, E., HALLINEN, T. & NISKANEN, L., 2008. Cost-effectiveness of statins in the prevention of coronary heart disease events in middle-aged Finnish men. *Current Medical Research and Opinion,* 24(6),pp.1823-1832
PHAROAH, P.D. & HOLLINGWORTH, W., 1996. Cost effectiveness of lowering cholesterol concentration with statins in patients with and without pre-existing coronary heart disease: life table method applied to health authority population. *BMJ,* 312(7044), pp. 1443-1448.
RAIKOU, M., MCGUIRE, A., COLHOUN, H.M., BETTERIDGE, D.J., DURRINGTON, P.N., HITMAN, G.A., NEIL, H.A.W., LIVINGSTONE, S.J., CHARLTON-MENYS, V. & FULLER, J.H., 2007. Cost-effectiveness of primary prevention of cardiovascular disease with atorvastatin in type 2 diabetes: results from the Collaborative Atorvastatin Diabetes Study (CARDS). *Diabetologia,* 50(4), pp. 733-740.
RAMSEY, S.D., CLARKE, L.D., ROBERTS, C.S., SULLIVAN, S.D., JOHNSON, S.J. & LIU, L.Z., 2008. An economic evaluation of atorvastatin for primary prevention of cardiovascular events in type 2 diabetes. *PharmacoEconomics,* 26(4), pp. 329-339.
ROSEN, V.M., TAYLOR, D.C., PAREKH, H., PANDYA, A., THOMPSON, D., KUZNIK, A., WATERS, D.D., DRUMMOND, M. & WEINSTEIN, M.C., 2010. Cost effectiveness of intensive lipid-lowering treatment for patients with congestive heart failure and coronary heart disease in the US. *PharmacoEconomics,* 28(1), pp. 47-60.
SCUFFHAM, P.A. & CHAPLIN, S., 2005. A cost-effectiveness analysis of fluvastatin in patients with diabetes after successful percutaneous coronary intervention. *Clinical therapeutics,* 27(9), pp. 1467-1477.

SCUFFHAM, P.A. & CHAPLIN, S., 2004. An economic evaluation of fluvastatin used for the prevention of cardiac events following successful first percutaneous coronary intervention in the UK. *PharmacoEconomics,* 22(8), pp. 525-535.

SCUFFHAM, P.A. & KOSA, J., 2006. The cost-effectiveness of fluvastatin in Hungary following successful percutaneous coronary intervention. *Cardiovascular Drugs & Therapy,* 20(4), pp. 309-317.

SIGVANT, B., HENRIKSSON, M., LUNDIN, F. & WAHLBERG, E., 2011. Asymptomatic peripheral arterial disease: is pharmacological prevention of cardiovascular risk cost-effective? *European journal of cardiovascular prevention and rehabilitation : official journal of the European Society of Cardiology, Working Groups on Epidemiology & Prevention and Cardiac Rehabilitation and Exercise Physiology,* 18(2), pp. 254-261.

SLEJKO, J.F., PAGE, R.L.,2ND & SULLIVAN, P.W., 2010. Cost-effectiveness of statin therapy for vascular event prevention in adults with elevated C-reactive protein: implications of JUPITER. *Current Medical Research & Opinion,* 26(10), pp. 2485-2497.

SOINI, E.J., DAVIES, G., MARTIKAINEN, J.A., HU, H.X., TUNCELI, K. & NISKANEN, L., 2010. Population-based health-economic evaluation of the secondary prevention of coronary heart disease in Finland. *Current Medical Research & Opinion,* 26(1), pp. 25-36.

SPAANS, J.N., COYLE, D., FODOR, G., NAIR, R., VAILLANCOURT, R., GROVER, S.A. & COUPAL, L., 2003. Application of the 1998 Canadian cholesterol guidelines to a military population: health benefits and cost effectiveness of improved cholesterol management. *Canadian Journal of Cardiology,* 19(7), pp. 790-796.

SZUCS, T.D., BERGER, K., MÄRZ, W. and SCHÄFER, J.R., 2000. Cost-effectiveness of pravastatin in secondary prevention in patients with myocardial infarction or instable angina in Germany. An analysis on the basis of the LIPID trial. *Herz,* 25(5), pp. 487-494.

SZUCS, T.D., BERTEL, O., DARIOLI, R., GUTZWILLER, F. & MORDASINI, R., 2000. Pharmacoeconomic evaluation of pravastatin in coronary secondary prevention in patients with myocardial infarct or unstable angina pectoris. An analysis based on the LIPID Study. *Praxis,* 89(18), pp. 745-752.

SZUCS, T.D., GUGGENBERGER, G., BERGER, K., MÄRZ, W. & SCHÄFER, J.R., 1998. Pharmacoeconomic evaluation of pravastatin in the secondary prevention of coronary heart disease in patients with average cholesterol levels. An analysis for Germany based on the CARE study. *Herz,* 23(5), pp. 319-329.

SZUCS, T.D., KLOSE, G. & DUSING, R., 2004. Cost-effectiveness of atorvastatin for the prevention of coronary disease. An analysis of the ASCOT study. *Deutsche Medizinische Wochenschrift,* 129(25-26), pp. 1420-1424.

TAYLOR, D.C.A., PANDYA, A., THOMPSON, D., CHU, P., GRAFF, J., SHEPHERD, J., WENGER, N., GRETEN, H., CARMENA, R., DRUMMOND, M. & WEINSTEIN, M.C., 2009. Cost-effectiveness of intensive atorvastatin therapy in secondary cardiovascular prevention in the United Kingdom, Spain, and Germany, based on the treating to new targets study. *European Journal of Health Economics,* 10(3), pp. 255-265.

TONKIN, A.M., ECKERMANN, S., WHITE, H., FRIEDLANDER, D., GLASZIOU, P., MAGNUS, P., KIRBY, A., MULRAY, S., DENTON, M., SALLABERGER, M., HUNT, D., SIMES, J. & LIPID STUDY, G., 2006. Cost-effectiveness of cholesterol-lowering therapy with pravastatin in patients with previous acute coronary syndromes aged 65 to 74 years compared with younger patients: results from the LIPID study. *American Heart Journal,* 151(6), pp. 1305-1312.

TROCHE, C.J., TACKE, J., HINZPETER, B., DANNER, M. & LAUTERBACH, K.W., 1998. Cost-effectiveness of primary and secondary prevention in cardiovascular diseases. *European Heart Journal,* 19(Suppl C), pp. 59-65.

TSEVAT, J., KUNTZ, K.M., ORAV, E.J., WEINSTEIN, M.C., SACKS, F.M. & GOLDMAN, L., 2001. Cost-effectiveness of pravastatin therapy for survivors of myocardial infarction with average cholesterol levels. *American Heart Journal,* 141(5), pp. 727-734.

VAN HOUT, B.A. & SIMOONS, M.L., 2001. Cost-effectiveness of HMG coenzyme reductase inhibitors; whom to treat?. *European Heart Journal,* 22(9), pp. 751-761.

WAGNER, M., LINDGREN, P., MERIKLE, E., GOETGHEBEUR, M. & JÖNSSON, B., 2009a. Economic evaluation of high-dose (80 mg/day) atorvastatin treatment compared with standard-dose (20 mg/day to 40 mg/day) simvastatin treatment in Canada based on the Incremental Decrease in End-Points Through Aggressive Lipid-Lowering (IDEAL) trial. *Canadian Journal of Cardiology,* 25(11), pp. e362-e369.

WAGNER, M., GOETGHEBEUR, M., MERIKLE, E., PANDYA, A., CHU, P. & TAYLOR, D.C., 2009b. Cost-effectiveness of intensive lipid lowering therapy with 80 mg of atorvastatin, versus 10 mg of atorvastatin, for secondary prevention of cardiovascular disease in Canada. *Canadian Journal of Clinical Pharmacology,* 16(2), pp. e331-45.

WARD S, LLOYD JONES M, PANDOR A, HOLMES M, ARA R, RYAN A, et al., 2007. Systematic review and economic evaluation of statins for the prevention of coronary events. *Health Technology Assessment*, 11(14)
